# Application of White Noise in Minors with Autism Spectrum Disorder

**DOI:** 10.3390/bs15070988

**Published:** 2025-07-21

**Authors:** Miquel Salmerón Medina, Ana Blázquez, Amanda Cercos, Rosa Calvo

**Affiliations:** 1 Department of Child and Adolescent Psychiatry and Psychology, Institute of Neuroscience, Hospital Clínic of Barcelona, 08036 Barcelona, Spain; 2 Child and Adolescent Psychiatry and Psychology Research Group, Institut d’Investigacions Biomèdiques August Pi i Sunyer (IDIBAPS), 08036 Barcelona, Spain; 3 Department of Basic Clinical Practice, Pharmacology Unit, University of Barcelona, 08036 Barcelona, Spain; 4 Department of Medicine, University of Barcelona, 08036 Barcelona, Spain; 5 Centro de Investigación Biomédica en Red de Salud Mental (CIBERSAM), Instituto de Salud Carlos III, 28029 Madrid, Spain

**Keywords:** anxiety, self-regulation, intellectual disability, auditory hyperreactivity, white noise, Autism Spectrum Disorder

## Abstract

Individuals with Autism Spectrum Disorder (ASD) often experience sensory hyperreactivities that interfere with daily life activities. White noise, characterized by its uniformity and its ability to mask environmental sounds, may serve as a tool to improve sensory and emotional regulation in children with ASD. The primary objective was to evaluate the response to white noise in improving self-regulation in minors with ASD. As a secondary objective, the study assessed whether there were differences in the response to white noise between patients with ASD and those with ASD and Intellectual Disability (ID). This study was conducted in the Child and Adolescent Psychiatry and Psychology Department of Hospital Clínic of Barcelona. A total of 54 patients, aged between 7 and 17 years, were included. The patients were divided into two groups: Group 1 consisted of patients diagnosed with ASD (*n* = 21), and Group 2 included patients diagnosed with ASD and ID (*n* = 33). White noise was offered to the patients, and their response was evaluated before and after the exposure using the Conners Teacher Rating Scale. Overall, the response to white noise in the sample was positive, with a significant difference in scores on the Conners Teacher Rating Scale (*p* < 0.001). When dividing the sample into the ASD group and the ASD + ID group, it was observed that the ASD + ID group tolerated white noise better and had a longer exposure time, although both groups showed improved scores on the Conners Teacher Rating Scale. White noise may be a valuable tool to enhance well-being in individuals with ASD, reduce motor restlessness, and increase attention span and emotional stability. However, its effectiveness varies across individuals. It is recommended to tailor its use to individual needs and to extend future research by incorporating physiological measures and larger sample sizes.

## 1. Introduction

Individuals with Autism Spectrum Disorder (ASD) may process environmental stimuli differently, often exhibiting sensory hyperreactivity—particularly to sound—which can lead to intense discomfort in response to certain auditory inputs (American Psychiatric Association, 1994, 2013; Graña, 2014). As a result, they may display atypical sensory responses to auditory stimuli (Chang et al., 2012), making them more sensitive to everyday sounds, even those that typically go unnoticed by most people. Sounds considered normal and tolerable for the majority may be overwhelming, uncomfortable, or even painful for individuals with ASD.

This heightened sensitivity to external stimuli often contributes to the emergence of anxiety and interferes with self-regulation abilities (Graña, 2014). This heightened sensitivity is better described as auditory hyperreactivity, referring to intense physiological and behavioral responses to everyday sounds, rather than altered perceptual thresholds. This distinction aligns with recent taxonomies of sensory processing in autism (He et al., 2023). Self-regulation can be influenced by how individuals process auditory stimuli, as suggested by psychoacoustic models that describe atypical responses to sound perception (Zwicker & Fastl, 2013). This capacity may be compromised in individuals with ASD.

White noise may have a calming effect on some individuals due to its consistent and uniform auditory properties. On the one hand, white noise can mask unwanted or disturbing environmental sounds, helping to reduce distractions and stress. By creating a stable and continuous auditory background, it enables the brain to focus more easily and feel less disrupted by unpredictable sounds. On the other hand, white noise provides auditory stability as it contains all frequencies in equal measure. Abrupt changes in auditory environments can be distressing for some individuals, whereas white noise offers a sense of continuity and predictability. Some individuals find it easier to relax and reduce intrusive thoughts by focusing on the repetitive and consistent properties of white noise, which may serve as a stabilizing auditory input (Johnson et al., 2010; Remington & Fairnie, 2017).

Several studies have identified sensory alterations as a core feature of Autism Spectrum Disorder (ASD), with auditory hyperreactivity being among the most prevalent and disabling manifestations (Johnson et al., 2010; Remington & Fairnie, 2017). This form of hyperreactivity is frequently linked to heightened stress levels and can significantly hinder engagement in daily activities such as attending school or participating in social interactions. Moreover, difficulties in sensory processing have been shown to directly affect emotional and behavioral self-regulation (Son & Kwag, 2021).

In this context, white noise has been explored as a potential sensory modulator. Its ability to provide a predictable auditory environment helps mask disruptive auditory stimuli, thereby facilitating a state of calm and reducing behavioral reactivity (Johnson et al., 2010). While tools such as the Conners Rating Scales-Revised (Conners, 1997) have been widely used to assess behavioral and attentional difficulties, recent studies have begun to explore how sensory strategies like white noise might complement such assessments and interventions.

Despite growing anecdotal and clinical evidence supporting the benefits of white noise in pediatric populations with ASD, few studies have systematically assessed its behavioral effects using standardized tools such as the Conners Teacher Rating Scale (Ghanizadeh & Mohammadi, 2008). This study aims to address that gap by providing systematically collected and quantifiable data.

The objectives of this study are to evaluate the effect of white noise on participants’ self-regulation capabilities, to examine its impact on attention and concentration, and to determine whether its use supports emotional and behavioral regulation. The study hypothesizes that white noise may serve as a sensory modulation strategy to reduce anxiety triggered by other sounds and have a beneficial effect on regulation in minors diagnosed with ASD by masking external stimuli, thereby facilitating greater control over their sensory environment.

## 2. Patients and Methods

This study was conducted in the Child and Adolescent Psychiatry and Psychology Department of Hospital Clínic de Barcelona between February 2023 and July 2024. The selected patients were treated at a specialized Autism Spectrum Disorder Reference Unit (URTEA). URTEA is a specialized outpatient reference unit providing interdisciplinary assessment and intensive treatment. It provides care for children and adolescents up to 18 years of age with highly complex Autism Spectrum Disorder presentations, including severe self-injurious behaviors, significant disruptive conduct, persistent communication barriers, and lack of response to previous interventions in other clinical or educational settings.

The unit offers various intervention modalities, such as weekly intensive visits and linkage to a day hospital, including home-based interventions and accompaniment. Additionally, children requiring hospital admission will be treated by a URTEA team member to ensure continuity in treatment approach and dynamics. The duration of the intervention typically ranges from 3 to 6 months.

Inclusion criteria require a formal diagnosis and a commitment from the family to actively participate in the intervention.

A total of 54 patients aged between 7 and 17 years were included, all diagnosed with autism according to DSM-5 criteria (Autism Spectrum Disorder) (American Psychiatric Association, 2013). Thirty-three of these patients also had a co-occurring diagnosis of Intellectual Disability (ID), defined according to the DSM-5 as an intelligence quotient (IQ) below 70 or deficits in adaptive functioning. The classification of Intellectual Disability was based on existing clinical documentation, including prior IQ test results and assessments of adaptive functioning recorded in the patients’ medical files. No new psychometric evaluations were administered during the study period. While unlikely, we acknowledge the possibility that the non-ID group may include borderline or undetected cases.

In a pre–post evaluation design, Son and Kwag (2021) assessed changes in motor restlessness, emotional regulation, and behavioral regulation in individuals with intellectual disabilities following exposure to white noise. Although their study did not focus exclusively on ASD, it employed an abbreviated version of the Conners Teacher Rating Scale (CTRS) to measure behavioral outcomes. This questionnaire consists of ten items grouped into three dimensions related to behavior and self-regulation:

- Attention problems (items 3, 9, and 10), which assess a person’s ability to maintain focus on tasks, follow instructions, and complete activities without distraction;
- Hyperactivity–impulsivity (items 1, 7, and 8), which examines the presence of motor restlessness, difficulty remaining still, and a tendency to act impulsively without considering consequences;
- Behavioral problems (items 2, 4, 5, and 6), which evaluate disruptive behavior, emotional dysregulation, and conflicts with peers or authority figures.

The questionnaires were completed by professionals in continuous contact with the patients, allowing for a comparative analysis before and after exposure to white noise.

The CTRS was selected due to its well-established psychometric properties, brevity, and ease of use in clinical settings. While CTRS has been widely used in ADHD research, its application in ASD populations is less common. However, the CTRS was deemed appropriate for this study as it assesses common behavioral domains such as attention problems, hyperactivity, and impulsivity, which are relevant to both ADHD and ASD. The CTRS was chosen primarily due to practical considerations, including time constraints and its proven reliability for assessing behavioral regulation in both populations.

The study was conducted in a controlled environment. Prior to exposure to white noise, participants engaged in play-based activities or interactions with professionals. When observable behaviors consistent with predefined criteria for nervousness appeared, a professional completed the Conners Questionnaire, and white noise was offered to the participant via headphones. Participants were then allowed to continue their activities or rest, depending on their individual needs. The duration of exposure varied according to each participant’s specific condition. The procedure ended when the patient removed the headphones and resumed the previous activity. Each participant received the intervention (white noise via headphones) on a single occasion during the study period. This methodological decision was made to better control experimental conditions and to enable a clearer pre–post comparison. In this study, the term “intervention” specifically refers to this single, discrete episode of white noise exposure, with the pre-intervention measurement conducted immediately before headphone placement, and the post-intervention measurement taken immediately after the participant removed the headphones. Immediately afterward, the Conners Questionnaire was completed again to assess potential changes. The decision to offer white noise was based on a predefined list of observable behaviors, such as increased motor activity, repetitive vocalizations, task avoidance, and signs of facial or postural agitation, used systematically by trained professionals. Although these criteria aimed to standardize the intervention, we acknowledge that all observational judgments carry a degree of subjectivity. For this reason, future research should complement behavioral observations with physiological measures.

Tolerance to white noise was operationalized as the participant’s ability to keep the headphones on for at least 30 s without showing immediate signs of rejection—such as removing them instantly, expressing intense discomfort, or displaying behaviors indicative of aversion. If a participant removed the headphones immediately after trying them, they were recorded as intolerant, and no meaningful exposure duration was registered.

Two comparison groups were established, participants diagnosed with ASD and those with ASD and ID, in order to explore potential differences in their responses to white noise.

White noise was delivered using circumaural headphones with passive noise cancelation (JBL Tune 500 model), connected to a Samsung Galaxy Tab A7 tablet running a white noise playback application. The track used had a duration of 20 min and included a frequency range spanning from 20 Hz to 20 kHz. The volume of the white noise was standardized on the device (medium level, previously calibrated) and could not be adjusted by the participant. This measure aimed to ensure consistency across all cases and avoid introducing variability in auditory input.

The intervention took place in an observation room with controlled environmental conditions: neutral lighting, absence of intense visual stimuli, and a constant temperature between 22 and 23 °C. Only one professional accompanied the participant during the session, in order to minimize external distractions. Most sessions were conducted in an individualized setting between the participant and a professional. However, in some cases, children shared space with peers during structured observation or therapy activities, which justifies the inclusion of items such as ‘conflict with peers’ in the behavioral assessment. Although most activities were semi-structured, professionals were instructed to observe and complete the rating scale based on the child’s general behavioral regulation, including their responses to prompts, transitions, and boundaries within the interaction. We acknowledge that some items on the scale may have limited contextual relevance in a non-instructional setting, and this has been noted as a limitation.

Only patients with a clinically confirmed diagnosis of ASD (with or without Intellectual Disability) who had provided informed consent were included in the study. Participants with severe psychiatric comorbidities (such as psychosis or acute bipolar disorder) or those who did not tolerate the use of headphones were excluded.

The study was approved by the ethics committee of Hospital Clínic de Barcelona. Informed consent was obtained from the participants and/or their legal guardians, ensuring compliance with ethical and legal standards in research. This study was conducted in accordance with the ethical principles set out in the 2020 Declaration of Helsinki, guaranteeing the protection of participants’ rights, safety, and well-being.

Data were analyzed using SPSS version 18.0 (SPSS Inc., Chicago, IL, USA). Descriptive statistics included percentages, means, and standard deviations. Parametric tests (Student’s *t*-test) were used to compare scores, with normality assessed through the Kolmogorov–Smirnov and Shapiro–Wilk tests, and variance homogeneity evaluated using Levene’s test. Statistical significance was set at *p* < 0.05.

## 3. Results

A total of 54 minors (*n* = 54) receiving care at URTEA participated in the study (Table 1). Participants were divided into two groups: one diagnosed with ASD (*n* = 21) and another with ASD and co-occurring Intellectual Disability (ASD + ID) (*n* = 33).

The mean age of participants was 11.22 years (SD = 3.04). The mean age within the ASD group was 13.28 years (SD = 1.87), while the ASD + ID group had a mean age of 9.90 years (SD = 2.92). Regarding sex distribution, no statistically significant differences were found between the two groups (*p* = 1.000).

When comparing the ASD group and the ASD + ID group, statistically significant differences were found, with the ASD + ID group being exposed to white noise for a longer duration (*p* = 0.004) (Table 1). Regarding tolerance to white noise, it was also better tolerated in the ASD + ID group (*p* = 0.000) (Table 1).

Table 2 presents the total scores obtained by participants on the Teacher’s Questionnaire by C. Keith Conners before and after the intervention, along with the corresponding means.

The mean scores from the questionnaire were compared before and after the intervention between the ASD group and the ASD + ID group. At the beginning of the intervention, statistically significant differences were found between the two groups, with *t* = 5.143 (*p* = 0.000). By the end of the intervention, however, the differences were no longer statistically significant (*t* = 1.271; *p* = 0.950).

Table 3 presents the mean scores of the participants across the three dimensions derived from the Conners Teacher Rating Scale. The data are differentiated by participant condition (ASD vs. ASD + ID).

The **attention problems** dimension showed a significant reduction in total scores following the intervention. The total score in the pre-intervention measurement was 243 points, which decreased to 65 points in the post-intervention measurement. When analyzing group-specific results, patients with ASD scored 87 points pre-intervention and 22 points post-intervention. In the ASD + ID group, the total score decreased from 156 to 43 points.

The **hyperactivity–impulsivity** dimension also showed a significant reduction in total scores after the intervention. The total pre-intervention score was 237 points, which dropped to 67 points post-intervention. Among patients with ASD, the score declined from 80 to 24 points, and in the ASD + ID group, from 157 to 43 points.

Finally, the **behavioral problems** dimension also revealed a significant decrease in total scores following the intervention. The total pre-intervention score was 229 points, while the post-intervention score was 55 points. In the ASD group, scores dropped from 75 to 15 points, and in the ASD + ID group, from 154 to 40 points.

## 4. Discussion and Conclusions

This study explored the effects of white noise in autistic minors, including those with autism and co-occurring intellectual disability, assessing both their tolerance to the stimulus and its impact on self-regulation and behavior. Results indicated that the ASD + ID group exhibited greater tolerance to white noise exposure compared to the ASD group. Following the intervention, both groups demonstrated significant improvements in scores on the Teacher’s Questionnaire by C. Keith Conners, particularly in the areas of attention, hyperactivity–impulsivity, and behavioral problems. Although significant differences were initially observed between the groups, these differences were no longer present at the end of the study, suggesting a general positive effect of white noise on behavioral and emotional regulation.

These results align with prior research addressing various aspects of auditory processing and behavioral regulation in individuals with ASD, including the role of white noise interventions, hyperreactivity to sound, and sleep-related difficulties (Conners, 1997; Ghanizadeh & Mohammadi, 2008; Gee et al., 2014; Torres & Denisova, 2016; Papadopoulos et al., 2019). In this study, the application of white noise in the ASD + ID group appeared to support emotional and behavioral regulation, potentially due to the reduction in disturbing environmental stimuli. The reduction in scores across all groups indicates a positive trend in symptom improvement.

Since the white noise intervention was applied during moments of observed agitation and was considered complete when the participant showed signs of regulation, it cannot be ruled out that the observed improvements reflect regression to the mean or a natural return to baseline. Therefore, although the results suggest an association between white noise exposure and improved self-regulation, it is not possible to conclude with certainty that this intervention was the direct cause of the observed change. This methodological aspect constitutes a key limitation of the study and should be taken into account when interpreting the results.

Specifically, the ASD + ID group demonstrated a marked reduction in motor restlessness, improved attention span, and showed fewer signs of distractibility. Additionally, signs of irritability diminished, and participants reached a more relaxed state. They were also less disruptive toward peers and responded more effectively to structured activities. These improvements are consistent with previous studies suggesting that white noise can have a calming effect, and may even improve sleep quality and reduce anxiety in individuals with intellectual disabilities (Afsharnejad et al., 2019). The 100% tolerance rate observed in the ASD + ID group may reflect greater sensory acceptance. However, an alternative interpretation could involve higher compliance or learned helplessness, particularly in individuals with limited communication abilities or greater conditioning to follow instructions. Future studies should explore this possibility using complementary assessment tools.

These findings reinforce the importance of adapting auditory environments to improve the well-being of individuals with ASD. Creating spaces with controlled auditory stimuli may contribute to a safer and more predictable environment for this population.

White noise appears to exert a calming effect for several reasons: on the one hand, by masking disruptive sounds and reducing distractions through a continuous background sound, and on the other, by providing a sense of continuity and predictability.

The findings of this study suggest the use of white noise as a complementary therapeutic tool in clinical, educational, and family settings. The significant reduction in scores across the domains of attention, hyperactivity–impulsivity, and behavior—observed in both the ASD and ASD + ID groups—suggests a genuine improvement in self-regulation and emotional stability.

In our discussion, we have expanded the comparison of our results with previous studies that report mixed or null effects in auditory interventions for Autism Spectrum Disorder (ASD). Some studies, such as those by Ghanizadeh and Mohammadi (2008) and Son and Kwag (2021), have found divergent results regarding the effectiveness of sensory auditory interventions, suggesting that factors such as the methodology used, sample heterogeneity, and the context of the intervention could influence the outcomes. Additionally, research by Gee et al. (2014) and Afsharnejad et al. (2019) has reported mixed results regarding the efficacy of white noise, with some participants showing improvements in emotional regulation, while others had no significant effects.

On the other hand, some recent studies have shown consistent positive effects when using white noise and other auditory stimuli to improve emotional regulation and reduce anxiety in individuals with ASD, which aligns with our findings. However, the lack of homogeneity in previous studies necessitates a more critical evaluation of the factors that may contribute to the effectiveness of these interventions. We believe that the variability observed in the results of the literature reflects the need to tailor interventions to the individual characteristics of participants, such as their sensory profiles and communication levels.

Our research, while promising, is positioned within this broader context, acknowledging that the efficacy of auditory modulation may depend on individual and contextual factors that are still poorly understood. Future studies should incorporate a broader range of sensory interventions and focus on the personal sensory profiles of participants to enhance the generalizability and understanding of the effects of auditory modulation in ASD.

From a clinical perspective, white noise could be integrated into classroom routines for students with special educational needs, incorporated into structured therapeutic activities, or used as support in family environments where sensory overload situations are common.

The higher tolerance and greater benefit observed in the ASD + ID group may be related to a different sensory processing threshold or to previous exposure to sensory regulation strategies. This hypothesis warrants further exploration in future research. Moreover, given that the sample included children with varying levels of functional communication, not all participants were able to verbally express discomfort. Future studies should consider using adapted interviews or preference assessments to explore this sensory dimension more thoroughly.

Moreover, given that the sample included children with varying levels of functional communication, not all participants were able to verbally express their discomfort. Future studies should consider using adapted interviews or preference assessments to explore this sensory dimension more thoroughly.

The fact that some participants did not tolerate the use of headphones may indicate that, rather than serving as a regulatory tool, white noise can be aversive for some autistic individuals, particularly those without intellectual disability. This potential negative effect should be carefully considered, as emphasizing benefits without acknowledging possible adverse effects could lead to inappropriate or even harmful interventions for certain individuals. Therefore, any implementation should be personalized and preceded by an individual sensory assessment.

Additionally, by limiting access to headphones only to moments when staff observed dysregulation, we may have restricted the autonomy of some participants who were capable of spontaneously requesting the intervention. Future studies could consider designs that incorporate active requests as an additional indicator to initiate the intervention.

The study has several strengths, including its clinical relevance and the use of pre- and post-exposure measurements, which allow for a quantitative assessment of the impact of white noise. However, certain limitations must also be acknowledged. The sample size may limit the generalizability of the findings, and individual variability in sensory sensitivity was not fully controlled.

An important limitation of the study is the potential variability between evaluators. While all evaluators were experienced clinicians, no formal inter-rater reliability measures were implemented. Future research should incorporate standardization procedures, such as joint training sessions or inter-rater reliability checks, to improve the consistency of behavioral ratings and reduce subjective interpretation.

One of the limitations of the study is the relatively small sample size and the unequal distribution between groups. These factors may limit the statistical power and generalizability of the results. Given the exploratory nature of this study and the ethical considerations of providing white noise only when participants exhibited signs of nervousness, no control group was included. The absence of a control group is a limitation that must be considered when interpreting the results. To address the potential effects of expectancy and spontaneous behavioral variation, a within-subject design was used, where each participant served as their own control. Additionally, the intervention was limited to a single episode to avoid bias from repeated exposures.

Future studies should aim to recruit larger and more balanced samples to strengthen the statistical power and improve the generalizability of the findings. Additionally, the inclusion of physiological measures—such as heart rate—obtained through standardized protocols could help assess the impact of white noise from a neurophysiological perspective, complementing behavioral data.

It would also be appropriate to design longitudinal studies to evaluate the persistence of observed effects over the medium and long term, as well as to compare the efficacy of white noise with other auditory stimuli, such as ambient music, natural sounds, or pink noise.

Another promising direction involves personalizing interventions based on each participant’s individual sensory profile, assessed using standardized tools such as the Sensory Profile 2.

Ultimately, the implementation of white noise as a therapeutic resource should be tailored to the specific needs of each individual, avoiding a one-size-fits-all approach. This study provides preliminary evidence of its usefulness as a support strategy for emotional and behavioral regulation in children and adolescents with ASD and ID, and opens new avenues toward more accessible, personalized, and non-invasive sensory interventions.

Future research should include a larger sample size and better control of individual and contextual variables. It would also be beneficial to incorporate physiological measures, such as heart rate, to complement observational data and provide a more precise analysis of white noise’s impact.

Based on the results, it is proposed that white noise be tailored to each individual’s auditory sensitivity, with exposure adjusted to their specific needs. Once benefits are identified in a controlled environment, its use could be extended to other contexts.

In conclusion, each person with ASD responds differently to sensory stimuli. While some may find white noise a calming and beneficial tool for concentration, others may perceive it as unpleasant or overwhelming. Therefore, the implementation of this strategy must consider each individual’s unique characteristics.

## Figures and Tables

**Table 1:** Demographic characteristics and response to exposure.

Variable	Total (*n* = 54)	ASD Group (*n* = 21)	ASD + ID Group (*n* = 33)	Statistic
Gender				
-Male	72.2% (*n* = 39)	71.4% (*n* = 15)	72.7% (*n* = 24)	χ^2^ = 0.011 *p* = 1.000
-Female	27.8% (*n* = 15)	28.6% (*n* = 6)	27.3% (*n* = 9)	
Mean Exposure Duration (minutes)	10.69 (SD = 11.00)	3.42 (SD = 4.89)	15.30 (SD = 11.39)	*t* = 4.519 *p* = 0.004 *
Tolerance to White Noise	83.3% (*n* = 45)	57.2% (*n* = 12)	100% (*n* = 33)	χ^2^ = 16.97 *p* = 0.000 *

* *p* statistical significance was set at *p* < 0.05.

**Table 2:** Descriptive pre- and post-intervention scores on the Teacher’s Questionnaire by C. Keith Conners.

	ASD Group (*n* = 21)	ASD + ID Group (*n* = 33)
**PRE**		
-Total	129 points	579 points
-Mean	6.14 (SD = 6.48)	17.55 (SD = 8.74)
**POST**		
-Total	54 points	123 points
-Mean	2.57 (SD = 3.37)	3.73 (SD = 3.18)
**Statistic**	*t* = 4.999, *p* = 0.000 *	*t* = 10.768, *p* = 0.000 *

* *p* statistical significance was set at *p* < 0.05.

**Table 3:** Dimensions of the Conners Teacher Rating Scale.

Group	Dimension	PRE Mean (SD)	POST Mean (SD)	*t*	*p*
**ASD**	Attention	0.77 (0.74)	0.33 (0.45)	5.676	<0.001
	Hyperactivity–Impulsivity	0.71 (0.71)	0.31 (0.36)	4.035	0.001
	Behavioral Problems	0.43 (0.66)	0.20 (0.33)	3.020	0.007
**ASD + ID**	Attention	1.96 (0.87)	0.45 (0.39)	11.067	<0.001
	Hyperactivity–Impulsivity	1.95 (1.01)	0.47 (0.38)	10.155	<0.001
	Behavioral Problems	1.50 (0.86)	0.27 (0.32)	8.785	<0.001

## Data Availability

The data are stored in corporate files of the Hospital Clínic of Barcelona and are accessible to authorized hospital staff.
